# Prevalence and clinical correlates of overweight and obesity among adults in Abha, Saudi Arabia: A retrospective cross-sectional study

**DOI:** 10.1097/MD.0000000000045804

**Published:** 2025-11-28

**Authors:** Awad Alsamghan, Syed Esam Mahmood, Ausaf Ahmad, Mohammed Abadi Alsaleem

**Affiliations:** a Department of Family and Community Medicine, College of Medicine, King Khalid University, Abha, Saudi Arabia; b Department of Community Medicine, Kalyan Singh Government Medical College, Bulandshahr, Uttar Pradesh, India.

**Keywords:** adult population, obesity, overweight, prevalence, Saudi Arabia

## Abstract

The global rise in overweight and obesity has become a critical public health issue, recognized by the World Health Organization as a leading cause of morbidity and mortality. In Saudi Arabia, alarming rates of obesity necessitate a closer examination of the baseline characteristics and clinical features associated with these conditions. This study aims to analyze the prevalence of overweight and obesity in an adult population in Abha, Saudi Arabia, and identify the contributing clinical factors. A retrospective review of health records from a specialized healthcare facility in Abha was conducted for the year 2024. Participants included adults aged 18 and older, with data on anthropometric measurements and relevant laboratory results analyzed to determine the prevalence of overweight and obesity. The body mass index (BMI) was calculated, and its association with clinical parameters was explored using logistic regression analysis. The study included 202 participants, revealing a mean age of 54.75 years and a mean BMI of 32.23 kg/m^2^, indicating a significant prevalence of obesity. Females represented a higher proportion of the obese population (77.6%), while obesity was prevalent across all age groups. Notably, higher rates of hypertension were observed in the obese cohort. Correlations between BMI and clinical parameters demonstrated significant associations between higher BMI, poorer glycemic control (haemoglobin A1c), and elevated fasting glucose levels. The analysis indicated significant predictors of obesity, including age, gender, glycemic control, and lipid profile abnormalities. The findings underscore the pressing need for targeted public health interventions aimed at addressing obesity and its associated health risks, particularly among females and older adults. This study contributes valuable insights into the clinical characteristics of individuals with obesity, emphasizing the importance of comprehensive approaches to managing obesity-related comorbidities in the Kingdom of Saudi Arabia.

## 1. Introduction

The prevalence of overweight and obesity has emerged as a significant public health concern globally, with the World Health Organization (WHO) identifying it as one of the leading causes of morbidity and mortality (WHO, 2021).^[1]^ The rising rates of overweight and obesity among adults are linked to a multitude of health complications, including cardiovascular diseases, diabetes, and certain types of cancer (Ng et al, 2014).^[2]^ Understanding the baseline characteristics and clinical features contributing to these conditions is essential for the development of effective prevention and intervention strategies.

Recent data indicate that obesity rates have tripled since 1975, with approximately 1.9 billion adults aged 18 years and older classified as overweight; of these, over 650 million are considered obese (WHO, 2021).^[1]^ In Saudi Arabia, the escalating prevalence of overweight and obesity is particularly concerning, with studies showing that around 35% of the adult population is classified as overweight and over 30% as obese (Alqarni, 2020; Al-Daghri et al, 2016).^[3,4]^

Several factors influence the prevalence of overweight and obesity, including genetic predisposition, dietary patterns, physical inactivity, and socio-economic status (Swinburn et al, 2019).^[5]^ The complexity of these interrelated factors necessitates a comprehensive examination of the baseline characteristics and clinical features of individuals affected by overweight and obesity.

Beyond individual health implications, the economic burden of obesity on healthcare systems is substantial, leading to increased medical costs and loss of productivity (Cawley and Meyerhoefer, 2012).^[6]^ This situation underscores the urgent need for targeted public health initiatives and policies aimed at mitigating the obesity epidemic. Therefore, this study aims to examine the prevalence of overweight and obesity in an adult population, with a focus on the baseline and clinical features that may contribute to these conditions. By identifying and analyzing these factors, we hope to provide insights that can inform future health interventions and policy decisions.

## 2. Methodology

### 2.1. Study design and setting

This retrospective study was conducted in Abha, a prominent hill station situated in the Sarawat Mountains of the Aseer region, Kingdom of Saudi Arabia. The investigation was carried out after receiving ethical clearance from the Institutional Ethics Committee at King Khalid University. Data collection was performed at a specialized private healthcare facility in Abha city, which primarily serves individuals with chronic diseases. This center caters not only to residents of Abha but also to patients from surrounding areas, underlining its significance as a regional healthcare hub.

The study entailed a retrospective review of annual health records maintained at the center, covering the period from January 2024 to December 2024. These records belonged to clients diagnosed with a range of chronic conditions, including diabetes mellitus, hypertension, cardiovascular diseases, and obesity. The objective was to identify trends in disease prevalence, treatment adherence, and clinical outcomes, thereby enhancing the understanding of chronic disease management practices in the region. By encompassing a variety of chronic health conditions, the study aimed to provide a holistic overview of the chronic disease landscape and inform future improvements in healthcare delivery and resource allocation.

### 2.2. Study population and sampling

Eligible participants included adult males and females aged 18 years and above, residing in Abha city or its adjacent areas. Trained personnel extracted data from the health records. The minimum required sample size was calculated using the formula:


$$\begin{array}{l} \;n=(Z){}^{2}\times p(1-p)/d{}^{2}, \end{array}$$

where *p* was assumed to be 50%, *Z* = 1.96 for a 95% confidence level, and *d* = 6.86% (margin of error), based on a population estimate of 100,000. This yielded a minimum sample size of 202 participants. This sample size ensures sufficient power to detect a 10% difference in obesity prevalence across gender and age groups, consistent with regional prevalence data.^[3]^

### 2.3. Clinical description of predictor and outcome variables

Anthropometric measurements and laboratory results were reviewed for each participant. Data were extracted by trained staff using standardized protocols to ensure consistency, with quality checks performed on 5% of records to confirm accuracy. Laboratory measurements followed standard protocols, with equipment calibrated daily to ensure reliability. body mass index (BMI) was calculated using the standard formula: weight (kg) divided by height (m^2^). Based on WHO classification,^[7]^ BMI was categorized as: Normal weight: 18.0 to 24.9 kg/m^2^; Overweight: 25.0 to 29.9 kg/m^2^; and Obese: ≥30.0 kg/m^2^.

Hypertension was defined according to established clinical guidelines as either systolic blood pressure ≥ 140 mm Hg, diastolic pressure ≥ 90 mm Hg, current use of antihypertensive medication, or a confirmed diagnosis in the medical record.^[8]^

### 2.4. Laboratory investigations and reference ranges^[9,10,11,12]^

Blood samples were collected and analyzed using standardized laboratory protocols. The following parameters were assessed: Glycaemic control: Haemoglobin A1c (HbA1c): Normal range = 4..0 to 5.6%; Serum fasting glucose: Normal range = 3.9 to 5.5 mmol/L; Lipid profile: Total cholesterol: <5.2 mmol/L; Triglycerides: <1.7 mmol/L; Low-density lipoprotein (LDL): <3.4 mmol/L; High-density lipoprotein (HDL): 1.0 to 2.0 mmol/L; Liver function tests: Aspartate aminotransferase (AST): 0 to 40 U/L; Alanine aminotransferase (ALT): 0 to 40 U/L; Alkaline phosphatase (ALP): 30 to 120 IU/L. Renal function: Serum creatinine: 44 to 80 µmol/L. Thyroid function tests: Thyroid-stimulating hormone (TSH): 0.4 to 4.5 µU/mL; Free triiodothyronine (FT3) and free thyroxine (FT4): Assessed against standard laboratory references; Vitamin D levels: Normal range = 30 to 50 ng/mL; Hematological parameters: Haemoglobin (Hb): Males: 13.0 to 17.0 g/dL; Females: 11.5 to 15.5 g/dL; Mean corpuscular volume (MCV) and platelet count: Evaluated according to standard laboratory ranges.

### 2.5. Data analysis

Statistical analysis was conducted using SPSS version 20. Patient characteristics (categorical variables) were reported as numbers and percentages. Missing data (<5% for most variables) were excluded from analyses, with sensitivity checks confirming minimal impact on results. Potential confounding variables, such as socioeconomic status, diet, and physical activity, were considered but not fully captured due to limited data availability in the retrospective records. Where available, these factors were assessed descriptively to explore their potential influence on obesity outcomes, with future studies recommended to incorporate comprehensive patient-reported data on these variables. The Chi-square test (χ^2^) or Fisher exact test was employed to assess differences in the prevalence of normal weight, overweight, and obesity, as well as baseline characteristics of patients. To address multiple comparisons, Bonferroni correction was applied (*P* < .002 for 25 tests) to minimize the risk of Type I errors. Variables significantly associated (*P* < .05) with different BMI categories were included in the analysis. Bivariate logistic regression analysis was utilized to determine associations between patient characteristics and overweight/obesity, providing unadjusted and adjusted *P*-values, odds ratios, and corresponding 95% confidence intervals. Variance inflation factors (VIF < 5) confirmed no multicollinearity.

## 3. Results

### 3.1. Association between gender, age, and BMI categories

Table 1 shows a significant association between gender and BMI categories (*P*-value = .001). Females are more likely to be obese (77.6%) compared to males (22.4%). This suggests that females are at a higher risk of obesity. There is no significant association between age groups and BMI categories (*P*-value = .544). This indicates that obesity is prevalent across all age groups.

**Table 1:** Association of baseline characteristics with BMI categories.

Baseline characteristics	BMI (kg/m^2^)						*P*-value
	Normal 18.0–24.9		Overweight 25.0–29.9		Obesity ≥30		
	N	%	N	%	N	%	
Gender							
Female	6	33.3	22	44.0	104	77.6	.001*
Male	12	66.7	28	56.0	30	22.4	
Age groups (yr)							
<44	6	33.3	10	20.0	42	31.3	.544**
44–64	6	33.3	20	40.0	52	38.8	
≥64	6	33.3	20	40.0	40	29.9	
Systolic blood pressure							
Hypertension (≥140 mm Hg)	4	22.2	16	32.0	52	38.8	.319
Normal	14	77.8	34	68.0	82	61.2	
Diastolic blood pressure							
Hypertension (≥90 mm Hg)	0	0.0	4	8.0	4	3.0	0.200**^,^***
Normal	18	100.0	46	92.0	130	97.0	

BMI = body mass index.

* χ^2^ significant at 0.05 level.

** >20% cells have expected counts < 5.

*** Minimum expected cell count < 1.

### 3.2. Blood pressure and BMI categories

The results also show that there is no significant association between systolic blood pressure hypertension and BMI categories (*P*-value = .319). However, the prevalence of systolic blood pressure hypertension is higher in the obese group (38.8%) compared to the normal weight group (22.2%). Similarly, there is no significant association between diastolic blood pressure hypertension and BMI categories (*P*-value = .200). The prevalence of diastolic blood pressure hypertension is low across all BMI categories (Table 1).

### 3.3. Implications of findings

The study’s findings highlight the importance of addressing obesity, particularly among females, to reduce the risk of related health complications. Additionally, the results suggest that obesity is a significant health concern across all age groups, emphasizing the need for comprehensive health interventions (Table 1).

### 3.4. Descriptive statistics of participants

The descriptive statistics presented in Table 2 provide an overview of the baseline characteristics of the study participants. The mean age of the participants is 54.75 years, with a standard deviation of 17.38 years. The age range is quite broad, spanning from 15 to 89 years, indicating a diverse sample. The mean BMI is 32.23 kg/m^2^, with a standard deviation of 6.40 kg/m^2^. The BMI range is also wide, from 14.51 to 64.83 kg/m^2^, suggesting a significant variation in body mass among participants. The mean systolic blood pressure is 136.35 mm Hg, with a standard deviation of 16.60 mm Hg. The systolic blood pressure range is 93.00 to 190.00 mm Hg, indicating a considerable variation in blood pressure among participants. The mean diastolic blood pressure is 72.95 mm Hg, with a standard deviation of 9.91 mm Hg. The diastolic blood pressure range is 51.00 to 94.00 mm Hg, suggesting a relatively narrower range compared to systolic blood pressure. Overall, the descriptive statistics suggest that the study participants have a high mean BMI, indicating a potential risk for obesity-related health issues.

**Table 2:** Descriptive statistics of baseline characteristics.

Baseline characteristics	N	Mean	Std. deviation	Low	High
Age (yr)	202	54.75	17.38	15.0	89.0
BMI (kg/m^2^)	202	32.23	6.40	14.51	64.83
Systolic BP (mm Hg)	202	136.35	16.60	93.00	190.00
Diastolic BP (mm Hg)	202	72.95	9.91	51.00	94.00

BMI = body mass index, BP = blood pressure.

### 3.5. Clinical characteristics of participants

The results of the clinical characteristics presented in Table 3 indicate several abnormalities. The mean HbA1C level is 8.63%, which is above the normal range (4.0–5.6%), indicating poorly controlled diabetes. The mean serum fasting glucose level is 9.26 mmol/L, which is above the normal range (3.9–5.5 mmol/L), further supporting the presence of diabetes. The lipid profile is also abnormal, with mean total cholesterol, triglyceride, and LDL levels of 4.36 mmol/L, 1.75 mmol/L, and 2.57 mmol/L, respectively, which are above the normal ranges (<5.2 mmol/L, <1.7 mmol/L, and <3.4 mmol/L, respectively). However, the mean HDL level is 1.10 mmol/L, which is within the normal range (1.0–2.0 mmol/L). Elevated levels of ASL, ALT, and ALP, with mean values of 22.23 U/L, 21.85 U/L, and 76.15 IU/L, respectively, which are above the normal ranges (0–40 U/L, 0–40 U/L, and 30–120 IU/L, respectively). The mean creatinine level is 75.31 µmol/L, which is within the normal range (44–80 µmol/L). The mean TSH level is 3.07 µU/mL, which is within the normal range (0.4–4.5 µU/mL). The mean FT3 and FT4 levels are also within normal limits. The mean vitamin D level is 19.54 ng/mL, which is below the normal range (30–50 ng/mL), indicating vitamin D deficiency or insufficiency. The mean Hb level is 13.89 g/dL, which is within the normal range (13.0–17.0 g/dL for males and 11.5–15.5 for females). The mean MCV and platelet count are also within normal limits. Overall, the clinical characteristics of the study participants indicate a high prevalence of diabetes, dyslipidemia, liver dysfunction, and vitamin D deficiency or insufficiency.

**Table 3:** Descriptive statistics of clinical characteristics.

Clinical characteristics	N	Mean	Std. deviation	Low	High
HbA1c (%)	202	8.63	2.07	4.70	14.90
Serum fasting glucose (mmol/L)	202	9.26	3.97	3.80	26.20
Total cholesterol (mmol/L)	202	4.36	1.15	1.50	9.64
Triglyceride (mmol/L)	202	1.75	1.27	0.22	8.66
HDL (mmol/L)	202	1.10	0.32	0.14	2.65
LDL (mmol/L)	202	2.57	0.86	0.22	6.58
ASL (U/L)	202	22.23	7.42	9.00	55.00
ALT (U/L)	202	21.85	10.29	9.00	58.00
ALP (IU/L)	202	76.15	26.44	34.00	185.00
Creatinine (µmol/L)	202	75.31	28.65	32.70	210.40
TSH (µU/mL)	202	3.07	1.99	0.36	10.98
FT3 (pg/mL)	202	4.98	0.64	3.00	7.17
FT4 (pmol/L)	202	9.62	1.40	6.53	13.67
Vitamin D (ng/mL)	202	19.54	7.24	2.30	45.90
Hb (g/dL)	202	13.89	2.10	8.30	19.50
MCV (fL)	202	84.60	9.54	26.20	98.00
Platelet (L)	202	291.64	89.63	77.00	547.00

ALP = alkaline phosphatase, ALT = alanine aminotransferase, FT3 = free triiodothyronine, FT4 = free thyroxine, Hb = haemoglobin, HbA1c = haemoglobin A1c, HDL = high-density lipoprotein, LDL = low-density lipoprotein, MCV = mean corpuscular volume, TSH = thyroid-stimulating hormone.

### 3.6. Correlation analysis between BMI and clinical characteristics

**Figure 1.**
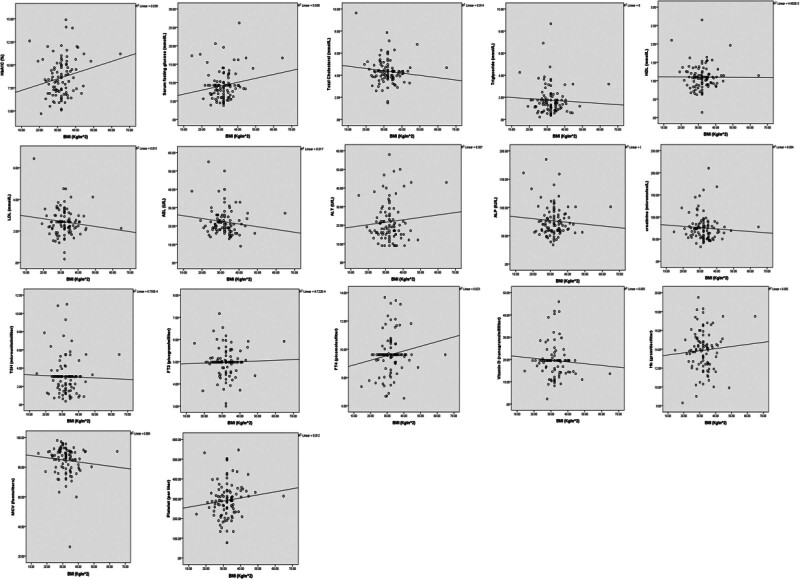
Scatter plot showing trend between BMI with clinical characteristics. BMI = body mass index.

Table 4 and Figure 1 show the correlation analysis between BMI and various clinical characteristics. Several significant relationships are observed. A significant positive correlation was found between BMI and HbA1c (%), indicating that higher BMI is associated with poorer glycemic control. Additionally, serum fasting glucose (mmol/L) also showed a significant positive correlation with BMI, suggesting that higher BMI is associated with higher fasting glucose levels. Furthermore, FT4 (pmol/L) showed a significant positive correlation with BMI, indicating that higher BMI is associated with higher FT4 levels. In contrast, a significant negative correlation was found between BMI and total cholesterol (mmol/L), indicating that higher BMI is associated with lower total cholesterol levels. Although not statistically significant, a negative correlation was also observed between BMI and LDL (mmol/L). No significant correlations were found between BMI and other clinical characteristics, including triglyceride (mmol/L), HDL (mmol/L), ASL (U/L), ALT (U/L), ALP (IU/L), creatinine (µmol/L), TSH (µU/mL), FT3 (pg/mL), vitamin D (ng/mL), Hb (gram/dL), MCV (fL), and platelet (per L). Overall, the results suggest that higher BMI is associated with poorer glycemic control, higher fasting glucose levels, and higher FT4 levels.

**Table 4:** Correlation of clinical characteristics with BMI.

Clinical characteristics	BMI (kg/m^2^)	
	*r*-value	*P*-value
HbA1c (%)	0.196	.005
Serum fasting glucose (mmol/L)	0.173*	.014
Total cholesterol (mmol/L)	−0.116	.014
Triglyceride (mmol/L)	−0.054	.442
HDL (mmol/L)	−0.007	.925
LDL (mmol/L)	−0.122	.083
ASL (U/L)	−0.129	.066
ALT (U/L)	0.082	.249
ALP (IU/L)	−0.078	.269
creatinine (µmol/L)	−0.066	.352
TSH (µU/mL)	−0.026	.714
FT3 (pg/mL)	0.031	.660
FT4 (pmol/L)	0.151*	.032
Vitamin D (ng/mL)	−0.071	.316
Hb (g/dL)	0.068	.333
MCV (fL)	−0.095	.178
Platelet (L)	0.111	.116

ALP = alkaline phosphatase, ALT = alanine aminotransferase, BMI = body mass index, FT3 = free triiodothyronine, FT4 = free thyroxine, Hb = haemoglobin, HbA1c = haemoglobin A1c, HDL = high-density lipoprotein, LDL = low-density lipoprotein, MCV = mean corpuscular volume, TSH = thyroid-stimulating hormone.

* Significant at 0.002 level (Bonferroni-adjusted).

### 3.7. Distribution of clinical and laboratory parameters across BMI categories

Table 5 shows the distribution of participants with various clinical and laboratory parameters across BMI categories. A higher proportion of participants in the overweight and obese categories had uncontrolled glycemia (>7.2 mmol/L) compared to those in the normal BMI category, although the difference was not statistically significant. The proportion of participants with high-risk triglyceride levels (>2.25) was higher in the overweight and obese categories, but not statistically significant. Conversely, a significantly higher proportion of participants in the normal BMI category had normal AST levels compared to overweight and obese categories. The thyroid function tests (TSH, FT3, FT4) did not show significant differences across BMI categories. Although not statistically significant, a higher proportion of participants in the overweight and obese categories had deficient or insufficient vitamin D levels. The haematological parameters revealed that a higher proportion of obese participants had low Hb levels, and a higher proportion of overweight participants had high platelet counts, with the difference in platelet counts being statistically significant. Overall, participants in the overweight and obese categories are more likely to have abnormal lipid profiles, liver function, and haematological parameters compared to those with normal BMI.

**Table 5:** Association of clinical characteristics with BMI categories.

Clinical characteristics	BMI (kg/m^2^)						*P*-value
	Normal (18.0–24.9)		Overweight (25.0–29.9)		Obesity (≥30)		
	n	%	n	%	n	%	
HbA1c							
Diabetes	14	77.8	44	88.0	114	85.1	.504*,†
Normal	2	11.1	2	4.0	4	3.0	
Prediabetes	2	11.1	4	8.0	16	11.9	
Serum fasting glucose							
Normal (<4.4 mmol/L)	0	0.0	2	4.0	2	1.5	.553*,†
Optimal glycaemic control (4.4–7.2 mmol/L)	4	22.2	18	36.0	42	31.3	
Uncontrolled glycaemia (>7.2 mmol/L)	14	77.8	30	60.0	90	67.2	
Total cholesterol							
Normal (<5.2 mmol/L)	14	77.8	42	84.0	112	83.6	.865*,†
Borderline (5.2–6.2 mmol/L)	2	11.1	6	12.0	14	10.4	
High risk (>6.2 mmol/L)	2	11.1	2	4.0	8	6.0	
Triglyceride							
Normal (<1.69)	10	55.6	24	48.0	86	64.2	.119*
Borderline (1.70–2.25 mmol/L)	6	33.3	12	24.0	20	14.9	
High risk (>2.25)	2	11.1	14	28.0	28	20.9	
HDL							
High	2	11.1	0	0.0	4	3.0	.008*,†
Low	2	11.1	20	40.0	28	20.9	
Normal	14	77.8	30	60.0	102	76.1	
LDL							
Borderline	2	11.1	10	20.0	12	9.0	.089*,†
High risk	2	11.1	0	0.0	8	6.0	
Normal	14	77.8	40	80.0	114	85.1	
AST							
High	2	11.1	0	0.0	2	1.5	.012*,†
Normal	16	88.9	50	100.0	132	98.5	
ALT							
High	0	0.0	2	4.0	8	6.0	.514*,†
Normal	18	100.0	48	96.0	126	94.0	
ALP							
High	2	11.1	2	4.0	2	1.5	.054*,†
Low	0	0.0	0	0.0	8	6.0	
Normal	16	88.9	48	96.0	124	92.5	
Creatinine							
High	4	22.2	10	20.0	12	9.0	.167*,†
Low	0	0.0	2	4.0	8	6.0	
Normal	14	77.8	38	76.0	114	85.1	
TSH							
High	4	22.2	8	16.0	26	19.4	.829*,†
Low	0	0.0	0	0.0	2	1.5	
Normal	14	77.8	42	84.0	106	79.1	
FT3							
Low	0	0.0	0	0.0	4	3.0	.355*,†
Normal	18	100.0	50	100.0	130	97.0	
FT4							
Low	6	33.3	14	28.0	26	19.4	.249
Normal	12	66.7	36	72.0	108	80.6	
Vitamin D							
Deficient	0	0.0	6	12.0	18	13.4	.200*
Insufficient	16	88.9	34	68.0	82	61.2	
Sufficient	2	11.1	10	20.0	34	25.4	
Hb							
High	0	0.0	10	20.0	10	7.5	.057*
Low	2	11.1	4	8.0	18	13.4	
Normal	16	88.9	36	72.0	106	79.1	
MCV							
Low	0	0.0	10	20.0	24	17.9	.128
Normal	18	100.0	40	80.0	110	82.1	
Platelet							
High	2	11.1	0	0.0	20	14.9	.040*,†
Low	0	0.0	2	4.0	8	6.0	
Normal	16	88.9	48	96.0	106	79.1	

ALP = alkaline phosphatase, ALT = alanine aminotransferase, AST = aspartate aminotransferase, BMI = body mass index, FT3 = free triiodothyronine, FT4 = free thyroxine, Hb = haemoglobin, HbA1c = hemoglobin A1c, HDL = high-density lipoprotein, LDL = low-density lipoprotein, MCV = mean corpuscular volume, TSH = thyroid-stimulating hormone.

* More than 20% cells have expected counts <5.

† Minimum expected cell count <1.

### 3.8. Predictors of overweight and obesity: logistic regression analysis

The binary logistic regression analysis in Table 6 identified several significant predictors of overweight and obesity (BMI levels). Age was a significant predictor, with participants in the 44 to 64 years age group being more likely to be overweight or obese. Males were more likely to be overweight or obese compared to females. Glycemic control was also a significant predictor, with participants having diabetes (A1C ≥ 6.5%) being more likely to be overweight or obese. Additionally, lipid profile factors, such as low LDL levels, were associated with increased likelihood of overweight and obesity. Other significant predictors included liver function (low ALP levels) and haematological parameters (low MCV and high platelet counts). These findings indicate that a combination of demographic, clinical, and laboratory factors contribute to the risk of overweight and obesity.

**Table 6:** Binary logistic regression analysis was used to find predictive value of baseline and clinical characteristics on dependent variable body mass index levels (overweight and obesity).

Baseline and clinical characteristics	*B*	SE	*P*-value	OR	95% CI for OR	
					Lower	Upper
Age groups (ref: <44 yr)			.05			
Age groups (44–64 yr)	−3.01	1.25	.02	.05	.00	.57
Age groups (≥64 yr)	−.69	.67	.31	.50	.14	1.87
Gender (ref: female) (male)	−1.34	.63	.03	.26	.08	.90
Systolic BP (ref: normal), hypertension (≥140 mm Hg)	.30	.68	.66	1.35	.35	5.13
Diastolic BP (ref: normal), hypertension (≥ 90 mm Hg)	.29	1.60	.86	1.33	.06	30.84
HbA1c (ref: normal)			.04			
HbA1c (prediabetes)	1.89	1.30	.15	6.64	.52	85.34
HbA1c (diabetes)	5.06	2.01	.01	158.12	3.07	8143.54
Serum fasting glucose (ref: normal [<4.4 mmol/L])			.26			
Serum fasting glucose (optimal glycaemic control [4.4–7.2 mmol/L])	21.17	6890.45	1.00	1.5 × 10^9^	.00	
Serum fasting glucose (uncontrolled glycaemia [>7.2 mmol/L])	1.17	.71	.10	3.21	.80	12.85
Total cholesterol (ref: normal [<5.2 mmol/L])			.71			
Total cholesterol (borderline [5.2–6.2 mmol/L])	−1.29	1.57	.41	.27	.01	5.99
Total cholesterol (high risk [>6.2 mmol/L])	20.15	28,420.72	1.00	5.8 × 10^8^	.00	
Triglyceride (ref: normal [<1.69])			.15			
Triglyceride (borderline [1.70–2.25 mmol/L])	1.35	.80	.09	3.87	.81	18.46
Triglyceride (high risk [> 2.25])	1.33	.91	.15	3.77	.63	22.43
HDL (ref: normal)			.18			
HDL (low)	−18.62	17,530.96	1.00	.00	.00	
HDL (high)	1.38	.74	.06	3.98	.93	17.01
LDL (ref: normal)			.02			
LDL (low)	5.09	1.86	.01	163.17	4.22	6308.90
LDL (high)	−39.88	30,958.47	1.00	.00	.00	
AST (ref: normal) (high)	−34.32	41,357.36	1.00	.00	.00	
ALT (ref: normal) (high)	.26	1.29	.84	1.30	.10	16.43
ALP (ref: normal)			.16			
ALP (low)	3.19	1.66	.05	24.27	.94	626.35
ALP (high)	−19.77	10,981.22	1.00	.00	.00	
Creatinine (ref: normal)			.15			
Creatinine (low)	−1.98	1.15	.08	.14	.01	1.31
Creatinine (high)	1.47	1.23	.23	4.35	.39	48.87
TSH (ref: normal)			.80			
TSH (high)	.18	.71	.80	1.20	.30	4.85
FT3 (ref: normal) (low)	13.32	30,044.87	1.00	611,675.84	.00	
FT4 (ref: normal) (low)	1.05	.70	.13	2.87	.73	11.30
Vitamin D (ref: deficient)			.76			
Vitamin D (insufficient)	.63	1.06	.55	1.88	.24	14.94
Vitamin D (sufficient)	−.11	.69	.88	.90	.23	3.45
Hb (ref: normal)			.95			
Hb (low)	.26	.96	.79	1.29	.20	8.49
Hb (high)	−.18	1.13	.87	.84	.09	7.67
MCV (ref: normal) (low)	1.64	.82	.04	5.17	1.05	25.58
Platelet (ref: normal)			.00			
Platelet (low)	−34.78	9744.57	1.00	.00	.00	
Platelet (high)	−4.94	1.43	.00*	.01	.00	.12
Constant	−3.15	1.76	.07	.04		

ALP = alkaline phosphatase, ALT = alanine aminotransferase, AST = aspartate aminotransferase, BP = blood pressure, CI = confidence interval, FT3 = free triiodothyronine, FT4 = free thyroxine, Hb = hemoglobin, HbA1c = haemoglobin A1c, HDL = high-density lipoprotein, LDL = low-density lipoprotein, MCV = mean corpuscular volume, OR = odds ratio, TSH = thyroid-stimulating hormone.

* Significant at .002 level (Bonferroni-adjusted).

## 4. Discussion

### 4.1. Obesity prevalence and contributing factors

Obesity is a significant global health concern, and understanding its prevalence and contributing factors is crucial for developing effective intervention strategies. A present study examined the prevalence of overweight and obesity in an adult population, revealing a significant association between gender and BMI categories, with females showing a higher likelihood of obesity compared to males. Obesity is prevalent across all age groups. Additionally, the prevalence of systolic hypertension was higher in the obese group compared to the normal weight group.

These findings align with existing literature on obesity prevalence and related risk factors. Several studies have reported gender differences in obesity prevalence, with women often exhibiting higher rates of obesity than men, as noted in a review by Kanter and Caballero (2012).^[13]^ Limited data on socioeconomic status and lifestyle factors (e.g., diet, physical activity) suggest these may influence obesity, warranting future studies with patient-reported outcomes.

This disparity may be attributed to various factors, including hormonal differences, sociocultural influences, and lifestyle behaviors.

The absence of a significant association between age and obesity in our study contrasts with some previous research. For instance, Ogden et al (2007) observed that the prevalence of overweight tends to increase with age.^[14]^ This discrepancy could be due to differences in the age distribution of the study population or variations in lifestyle factors across different age cohorts.

The relationship between obesity and hypertension is well-established. While our study did not find a statistically significant association between hypertension and BMI categories, the higher prevalence of systolic hypertension in the obese group is consistent with numerous studies linking obesity to increased blood pressure (Jiang et al, 2016).^[15]^

Obesity-induced hypertension is thought to be mediated by various mechanisms, including increased sympathetic nervous system activity, activation of the renin-angiotensin-aldosterone system, and insulin resistance (Kotsis et al, 2010).^[16]^

Overall, our study’s findings contribute to the existing body of evidence on obesity prevalence and associated health risks.

### 4.2. Baseline and clinical characteristics in an adult population with overweight and obesity

The study participants had a mean age of 54 years and exhibited various health issues, including obesity, elevated blood pressure, and a high prevalence of diabetes, dyslipidemia, and liver dysfunction. They also had vitamin D deficiency or insufficiency, abnormal lipid profiles, and high blood glucose levels, indicating a significant health burden.

These findings are consistent with existing literature on the clinical profiles associated with overweight and obesity. The mean age and BMI values reported in our study are comparable to those observed in other studies examining similar populations. Diabetes Prevention Program Research Group (2000) reported a mean BMI of 34 kg/m^2^ among participants at baseline.^[17]^

The elevated HbA1c and serum fasting glucose levels in our study indicate a high prevalence of poorly controlled diabetes, which is a common comorbidity of obesity. Dyslipidemia, characterized by abnormal lipid profiles, is also frequently observed in individuals with obesity. The elevated levels of total cholesterol, triglycerides, and LDL in our study are consistent with this pattern.

The finding of vitamin D deficiency or insufficiency is particularly noteworthy. Vitamin D deficiency has been increasingly recognized as a common issue in obese populations. Alloubani et al (2019) found that vitamin D levels were lowest in obese participants compared to overweight and normal-weight individuals.^[18]^ Vitamin D deficiency in obesity may be attributed to several factors, including reduced sun exposure, increased sequestration of vitamin D in adipose tissue, and impaired vitamin D metabolism.

The elevated levels of liver enzymes (ASL, ALT, and ALP) in our study suggest liver dysfunction, which may be indicative of nonalcoholic fatty liver disease (NAFLD). NAFLD is a common complication of obesity and is characterized by the accumulation of fat in the liver. Cimini et al (2017) discuss the relationship between adipose tissue dysfunction, vitamin D deficiency, and the pathogenesis of NAFLD.^[19]^

The baseline and clinical characteristics of the study participants indicate a high-risk population with a significant burden of obesity-related comorbidities. Present study highlight the importance of comprehensive interventions targeting diabetes, dyslipidemia, liver dysfunction, and vitamin D deficiency in individuals with overweight and obesity.

### 4.3. Correlations between BMI and clinical characteristics

Present study revealed a significant positive correlation between BMI and HbA1c (%), serum fasting glucose (mmol/L), and FT4 (pmol/L). Conversely, a significant negative correlation was observed between BMI and total cholesterol (mmol/L).

The positive correlation between BMI and HbA1c aligns with numerous studies demonstrating that higher BMI is associated with poorer glycemic control. Babikr et al (2016) found a significant positive correlation between HbA1c and BMI in type 2 diabetic patients.^[20]^ This relationship is likely mediated by insulin resistance, which is commonly observed in individuals with obesity.

Similarly, the positive correlation between BMI and serum fasting glucose is consistent with previous research. Koca (2017) reported a positive association between BMI and fasting glucose levels. This finding further supports the link between obesity and impaired glucose metabolism.^[21]^

The positive correlation between BMI and FT4 is an interesting finding. Mele et al (2022) observed a similar trend of increasing FT4 levels with increasing BMI in a large cohort of euthyroid patients with obesity.^[22]^ The underlying mechanisms for this association are not fully understood but may involve alterations in thyroid hormone metabolism or thyroid gland stimulation due to obesity-related factors.

The negative correlation between BMI and total cholesterol is somewhat unexpected, as many studies have reported positive associations between BMI and unfavorable lipid profiles. However, some studies have also reported negative or no significant correlations between BMI and total cholesterol. Flegal (2000) noted that the relationship between BMI and high blood cholesterol varies with age.^[23]^

Overall, our study’s findings provide complex relationships between BMI and various clinical characteristics. The positive correlations between BMI and HbA1c, serum fasting glucose, and FT4 highlight the metabolic consequences of obesity.

### 4.4. Clinical characteristics across BMI categories

A present study highlighted variations in glycemic control, lipid profile, liver function, thyroid function, vitamin D levels, and hematological parameters among normal weight, overweight, and obese participants.

The study observed a higher proportion of participants with uncontrolled glycemia and high-risk triglyceride levels in the overweight and obese categories, although these differences were not statistically significant. This aligns with Boye et al (2021), who investigated the relationship between obesity classes and glycemic control.^[24]^

A significant finding was the higher proportion of participants with low HDL levels in the overweight category. This is consistent with Rashid et al (2007), who examined the effect of obesity on HDL metabolism and noted decreased HDL levels in obese individuals.^[25]^

The study revealed a statistically significant difference in AST levels across BMI categories, with a higher proportion of normal AST levels in the normal BMI category. This finding supports Li et al (2020), who demonstrated that obesity predicts liver function testing and abnormal liver results.^[26]^

No significant differences were observed in thyroid function tests (TSH, FT3, FT4) across BMI categories. This is interesting, as Laurberg et al (2012) suggest that the pattern of thyroid function tests depends on the balance of obesity and underlying thyroid disease.^[27]^

Vitamin D Levels with BMI, although showed not statistically significant, However a higher proportion of participants in the overweight and obese categories had deficient or insufficient vitamin D levels. This observation is supported by Muscogiuri et al (2019), who reported a higher prevalence of vitamin D deficiency in subjects with obesity.^[28]^

The study found a statistically significant difference in platelet counts, with a higher proportion of high platelet counts in the overweight category. Additionally, a higher proportion of participants in the obese category had low hemoglobin levels. This is consistent with Purdy and Shatzel (2021), who noted hematologic consequences of obesity, including variations in leukocyte and platelet counts.^[29]^

While not all differences were statistically significant, the findings suggest that overweight and obese individuals may be at higher risk for metabolic, hepatic, and hematological abnormalities.

## 5. Future considerations

While our analysis identified key predictors of overweight and obesity, exploring potential interactions such as between demographic and clinical variables could reveal synergistic effects and improve understanding. Future models incorporating interaction terms may provide more nuanced insights. Regarding multicollinearity, VIFs were below 5, indicating minimal concern. However, examining correlations among predictors and using techniques like PCA or regularization could further validate the robustness of our findings. Overall, considering interactions and addressing collinearity can strengthen the interpretation and clinical relevance of our results.

## 6. Study limitation

Its retrospective design limits causal inference, as data were drawn from existing health records that might not include all relevant factors influencing patient outcomes. Selection bias stemming from the specialized healthcare setting may limit the generalizability of our findings to healthier populations and we recommend that future studies include more diverse populations to enhance applicability. Missing data (<5%) were excluded, with minimal impact on results. Confounding variables like socioeconomic status and lifestyle were not fully captured, and longitudinal studies are needed to explore causality. The Bonferroni correction (*P* < .002) may have reduced sensitivity for smaller effects. Also, cultural and regional differences may also affect the applicability of the findings to other areas.

## 7. Conclusion

This study discloses a concerning obesity prevalence among participants, with females being disproportionately affected. The association between higher BMI and adverse health outcomes, including hypertension and poor glycemic control, highlights significant health risks. Targeted interventions are urgently needed, considering age and gender factors, to address this growing public health challenge. Future longitudinal studies and inclusion of patient-reported outcomes and lifestyle factors can enhance understanding of obesity’s causes and effects. To better elucidate cause-and-effect relationships, future research employing longitudinal designs such as prospective cohort studies would be valuable. These approaches can track changes over time and provide stronger evidence of causal links.

To tackle the obesity epidemic and its health risks, recommendations include:

1. Implement community-based health programs focusing on lifestyle changes for women and older adults.
2. Encourage regular health screenings for hypertension, glycemic control, and lipid profiles in individuals with high BMI.
3. Develop educational campaigns to raise awareness about obesity risks.
4. Foster collaboration among healthcare providers for comprehensive care.
5. Advocate for policies promoting healthier food environments and increased physical activity.
6. Conduct longitudinal studies on obesity’s long-term effects and assess intervention strategies across demographics.

These efforts aim to promote sustainable change and enhance overall population health and well-being.

## Acknowledgments

We want to thank the study participants for participating.

## Author contributions

**Conceptualization:** Awad Alsamghan.

**Data curation:** Awad Alsamghan, Ausaf Ahmad.

**Formal analysis:** Ausaf Ahmad.

**Funding acquisition:** Awad Alsamghan.

**Investigation:** Awad Alsamghan.

**Methodology:** Syed Esam Mahmood, Mohammed Abadi Alsaleem.

**Project administration:** Awad Alsamghan.

**Resources:** Awad Alsamghan.

**Software:** Ausaf Ahmad.

**Supervision:** Awad Alsamghan.

**Validation:** Syed Esam Mahmood.

**Visualization:** Syed Esam Mahmood.

**Writing – original draft:** Syed Esam Mahmood.

**Writing – review & editing:** Awad Alsamghan, Syed Esam Mahmood, Ausaf Ahmad, Mohammed Abadi Alsaleem.
